# Evolutionary transitions in body plan and reproductive mode alter maintenance metabolism in squamates

**DOI:** 10.1186/s12862-018-1166-5

**Published:** 2018-04-03

**Authors:** Lin Zhang, Kun Guo, Guang-Zheng Zhang, Long-Hui Lin, Xiang Ji

**Affiliations:** 1 Jiangsu Key Laboratory for Biodiversity and Biotechnology, College of Life Sciences, Nanjing Normal University, Nanjing, Jiangsu 210023 China; 2 Hangzhou Key Laboratory for Animal Adaptation and Evolution, College of Life and Environmental Sciences, Hangzhou Normal University, Hangzhou Zhengjiang, 310036 China

**Keywords:** Body plan, Maintenance metabolism, Reproductive mode, Squamates, Standard metabolic rate

## Abstract

**Background:**

Energy (resources) acquired by animals should be allocated towards competing demands, maintenance, growth, reproduction and fat storage. Reproduction has the second lowest priority in energy allocation and only is allowed after meeting the energetic demands for maintenance and growth. This hierarchical allocation of energy suggests the hypothesis that species or taxa with high maintenance costs would be less likely to invest more energy in reproduction or to evolve an energetically more expensive mode of reproduction. Here, we used data on standard metabolic rate so far reported for 196 species of squamates to test this hypothesis.

**Results:**

We found that maintenance costs were lower in snakes than in lizards, and that the costs were lower in viviparous species than in oviparous species. As snakes generally invest more energy per reproductive episode than lizards, and viviparity is an energetically more expensive mode of reproduction than oviparity, our results are consistent with the hypothesis tested.

**Conclusion:**

The transition from lizard-like to snake-like body form and the transition from oviparity to viviparity are major evolutionary transitions in vertebrates, which likely alter many aspects of biology of the organisms involved. Our study is the first to demonstrate that evolutionary transitions in body plan and reproductive mode alter maintenance metabolism in squamates.

**Electronic supplementary material:**

The online version of this article (10.1186/s12862-018-1166-5) contains supplementary material, which is available to authorized users.

## Background

Metabolism is one of the most fundamental biological processes, encompassing all of the reactions catalyzed by enzymes to generate and use energy; metabolic rate is the most fundamental biological rate because it is the rate of energy uptake, transformation and allocation and, as such, is integrally related to the pace of life [1, 2]. In the case of animals, energy acquired by them will be allocated between two main competing demands, maintenance and production. Maintenance costs include the energy costs for basal (for endotherms) or standard (for ectotherms) metabolism and other activities essential for the continuity of an individual’s life, and energy allocated to production hierarchically supports somatic tissue growth, reproduction and fat storage [3–6]. Reproduction is not essential for the survival of an individual organism but it is important to reproduce for individual fitness and the continuity of populations and species. Earlier studies have showed that reproduction has the second lowest priority in energy allocation and only is allowed after the fulfillment of energetic requirements for maintenance and growth [6–10]. This hierarchical allocation of energy, when coupled with the fact that low energy costs for maintenance can translate into an increased potential to allocate more energy in reproduction, suggests the hypothesis that species or taxa with high maintenance costs would be less likely to invest more energy in reproduction or to evolve an energetically expensive mode of reproduction. To test this hypothesis, one needs to compare maintenance costs between animals that differ in reproductive investment or reproductive mode, best by means of using a group of animals spanning a relatively wide range of life-history and ecological strategies so it will be possible to draw some general conclusions.

Here, we used standard metabolic rate (SMR) data so far reported for 196 species of squamates, of which 128 (80 lizards and 48 snakes) are oviparous and 68 (35 lizards and 33 snakes) are viviparous (Additional file 1: Table S1), to test the above hypothesis. When compared to lizards, snakes generally invest more energy in reproduction per episode and thus have a relatively higher clutch mass [11–13]. Viviparity is an energetically expensive mode of reproduction not only because the physiological cost of supporting a litter is an important component of reproductive effort, but also because a shift in thermal ecology (longer basking periods and higher body temperatures) may elevate maternal metabolism, provoke postpartum emaciation and increase exposure of females to predators [14–16]. Thus, if the above hypothesis is true, it should follow that maintenance costs are lower in snakes than in lizards, and that the costs are lower in viviparous species than in oviparous species.

## Methods

We performed a bibliographic search for data on SMR, body mass and temperature (at which SMR was measured) reported for 196 species of squamate reptiles (115 lizards of the families Agamidae, Anniellidae, Anguidae, Cordylidae, Crotaphytidae, Dactyloidae, Gekkonidae, Iguanidae, Lacertidae, Liolaemidae, Phrynosomatidae, Pygopodidae, Scincidae, Sphaerodactylidae, Teiidae, Varanidae and Xantusiidae, and 81 snakes of the families Acrochordiae, Boidae, Colubridae, Elapidae, Pythonidae and Viperidae). To do that, we searched for references as thoroughly as possible from the Google Scholar and Web of Science, using the following keywords, lizard, metabolic rate, reptile, resting metabolic rate, squamate, standard metabolic rate and snake. We made great efforts to ensure that: (1) animals were fasted, inactive and measured at temperatures allowing normal activity but not during the breeding season (thus not allowing to address sex-specific rates in individual species); (2) thermal acclimation under laboratory conditions was brief; and (3) only data from the most well designed experiment were used in the case that more than one dataset was available for a species. We used the Reptile Database (www.reptile-database.org, accessed November 20, 2017) to check species name, excluding the data from synonyms and including the data from species risen from subspecies. Our dataset covered body masses from 0.4 g (*Sphaerodactylus beattyi*) to 16,150 g (*Python sebae*), and temperatures from 20 °C to 40 °C (Additional file 1: Table S1).

All data were transformed to natural logarithms prior to further analyses. We followed a method described by Brown et al. [1] to calculate temperature-corrected SMR [tSMR = ln(B_0_) + *b* lnM] and mass-corrected SMR [mSMR = ln(B_0_) − E(1/kT)], where B_0_ is a normalization constant independent of body size and temperature, *b* is the allometric exponent, M is body mass, E is the activation energy, k is the Boltzmann’s constant, and T is absolute temperature in K, thereby controlling both temperature and body mass and getting the residual SMR. We used both ordinary least squares (OLS) and phylogenetic generalized least squares (PGLS) regressions implemented in R 3.3.0 [17] with the packages *rms* [18] and *caper* [19] to test whether relationships between selected pairs of variables were significant. The Akaike’s Information Criterion (AIC) and the likelihood-ratio test (LRT) were used to assess the adequacy of models tested [20]. We performed PGLS to account for the non-independence of the data due to the shared evolutionary history of species. To do that, we constructed a phylogeny for the 196 species using Mesquite 3.04 [21] based on the relations at the species-level [22]. We followed a method described by Martins and Garland [23] to set branch lengths to 1 and used Mesquite 3.04 to reconstruct the ancestral states for the traits analyzed. Phylogenetic signal was measured by Pagel’s lambda (λ). Lambda values of or near 0 indicate phylogenetic independence; λ values of or near 1 indicate that the variable is fully explained by evolutionary history and thus shows the maximal strength of phylogenetic signal [24, 25]. The results from the phylogeny with variable branch lengths [26] were the same as those from the phylogeny with equal branch lengths (Additional file 2: Tables S2, Additional file 3: Table S3, Additional file 4: Table S4, Additional file 5: Table S5, Additional file 6: Table S6). Therefore, we present the results using equal branch lengths so as to include 25 species of which sequences for reconstructing the phylogeny were either unavailable or incomplete. We used mSMR or tSMR as the response variable, and reproductive mode (oviparity versus viviparity), animal clade (lizards versus snakes) and temperature (for mSMR) or body mass (for tSMR) as the explanatory variables to test if the two response variables differed between oviparous and viviparous species and between lizards and snakes.

To better explore directionality between transitions in reproductive mode or body plan and maintenance metabolism, we performed phylogenetic confirmatory path analyses [27] based on nine candidate path models in R 3.3.0 [17] with the package *phylopath* [28]. We ranked all candidate models based on their C-statistic Information Criterion (CICc) and selected the best model with ΔCICc ≤2 [27].

## Results

**Fig. 1:**
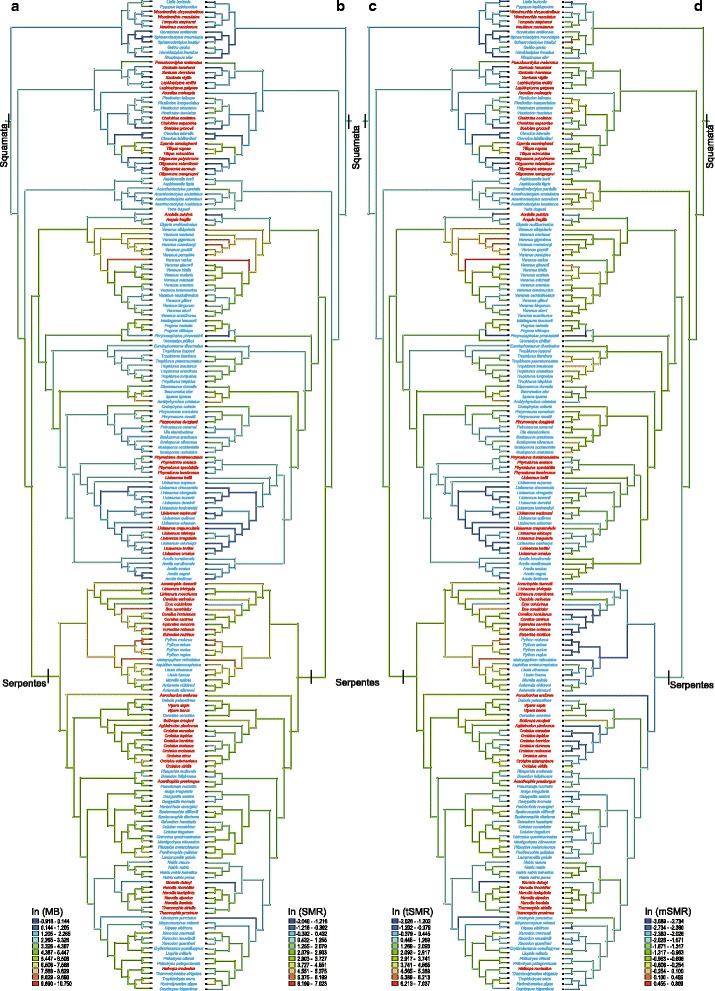
Mirror trees of the evolutionary history reconstructions of body mass (**a**), standard metabolic rate (**b**), temperature-corrected standard metabolic rate (**c**) and mass-corrected standard metabolic rate (**d**). Oviparous species are in blue, and viviparous species in red. Ancestral states are shown for visual purposes

**Fig. 2:**
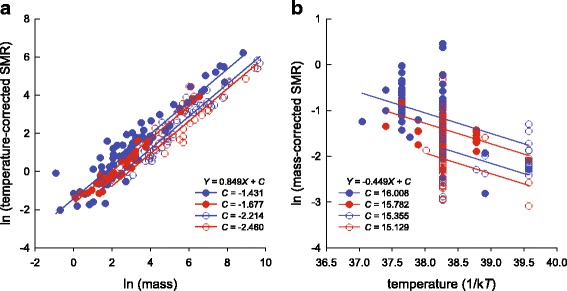
Metabolic rates of squamate reptiles as a function of body mass (**a**) or temperature (**b**). Lines represent PGLS regressions. Filled circles: lizards; open circles: snakes; red line: viviparous species; blue line: oviparous species

Our reconstruction of evolutionary changes in metabolic variables shows strong positive relationships between metabolic rate and body mass (SMR: *r*^2^ = 0.88, *F*_1, 194_ = 1410.91, *P* <  0.0001; tSMR: *r*^2^ = 0.88, *F*_1, 194_ = 1407.94, *P* <  0.0001), and that the viviparous taxa generally have lower mSMRs than the oviparous taxa (*t* = 2.461, *df* = 194, *P* = 0.015) (Fig. 1). The PGLS model provided a better fit to tSRM or mSMR than the OLS model, as determined by AIC and LRT (Table 1). We therefore used the PGLS model to analyze data. PGLS analysis on tSMR revealed that: 1) the effects of reproductive mode, animal clade and body mass are significant; 2) the mean tSMR is higher in the oviparous taxa than in the viviparous taxa, and is higher in lizards than in snakes; and 3) one of the four interactions is significant (Table 2, Fig. 2a). PGLS analysis on mSMR revealed that: 1) the effects of reproductive mode, animal clade and temperature are significant; 2) the mean mSMR is higher in oviparous species than in viviparous species, and is higher in lizards than in snakes; and 3) none of the four interactions is significant (Table 3, Fig. 2b).

**Table 1:** Statistics describing the relationships shown in Fig. 1. Models were fitted using both OLS and PGLS regressions

Model	*N*	Slope (±SE)	*r* ^2^	λ (95%CI)	AIC	ln likelihood	*F*_1, 194_	*P*-value
tSMR vs body mass								
OLS	196	0.85 (0.02)^**^	0.91		316.2	−153.5	1407.97	< 0.001
	196	0.76 (0.02)^**^	0.88	0	314.6	−151.3	1408.00	< 0.001
PGLS	196	0.85 (0.03)^**^	0.86	0.61 (0.51/0.91)	308.8	−148.4^a^	1113.50	< 0.001
	196	0.87 (0.03)^**^	0.79	1	312.9	−150.5	716.21	< 0.001
mSMR vs temperature								
OLS	196	−0.42 (0.08)^**^	0.41		311.8	−150.9	21.97	< 0.001
	196	−0.47 (0.08)^**^	0.24	0	309.8	−148.9	61.57	< 0.001
PGLS	196	−0.45 (0.09)^**^	0.16	0.51 (0.39/0.87)	293.8	−140.9^a^	23.09	< 0.001
	196	−0.38 (0.10)^**^	0.17	1	312.7	−150.4	14.97	< 0.001

*CI* confidence interval

^a^ the PGLS model is significantly better than the OLS model (likelihood ratio test)

^**^ *P* <  0.0001

**Table 2:** ANOVA output of the PGLS model {ln(tSMR) ~ R × G × ln(M)} evaluating the effects of reproductive mode (R), animal clade (G) and body mass (M) on temperature-corrected standard metabolic rate (tSMR)

Source	*df*	SQ	MSQ	*F*	*P*-value
Reproductive mode	1	0.034	0.034	24.47	< 0.001
Animal clade	1	0.079	0.079	59.60	< 0.001
Body mass	1	2.859	2.859	2055.56	< 0.001
R × G	1	0.012	0.012	8.31	0.004
R × M	1	0.002	0.002	1.64	0.202
G × M	1	0.003	0.003	2.63	0.126
R × G × M	1	0.002	0.002	1.55	0.215
Residuals	188	0.261	0.001

**Table 3:** ANOVA output of the PGLS model {ln(mSMR) ~ R × G × T} evaluating the effects of reproductive mode (R), animal clade (G) and temperature (T) on mass-corrected standard metabolic rate (mSMR)

Source	*df*	SQ	MSQ	*F*	*P*-value
Reproductive mode	1	0.014	0.014	7.32	0.007
Animal group	1	0.007	0.007	3.49	0.063
Temperature	1	0.056	0.056	29.61	< 0.001
R × G	1	0.005	0.005	2.80	0.096
R × T	1	0.002	0.002	0.93	0.335
G × T	1	< 0.001	< 0.001	0.25	0.621
R × G × T	1	0.003	0.003	1.62	0.205
Residuals	188	0.358	0.002		

**Fig. 3:**
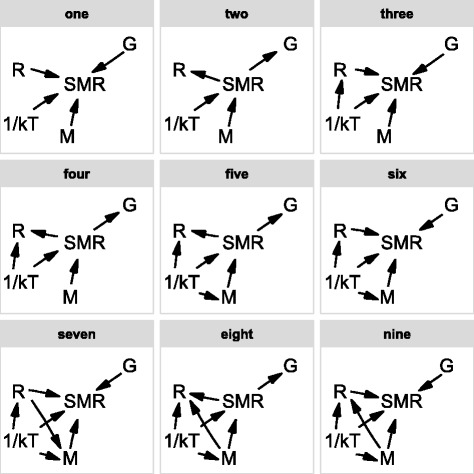
Directed acyclic graphs representing nine candidate models compared to disentangle the relationships between five traits through phylogenetic confirmatory path analyses and multi-model inference. M = body mass; SMR = standard metabolic rate; G = animal clade (lizards versus snakes); R = reproductive mode (oviparity versus viviparity); 1/kT = 1/temperature

**Fig. 4:**
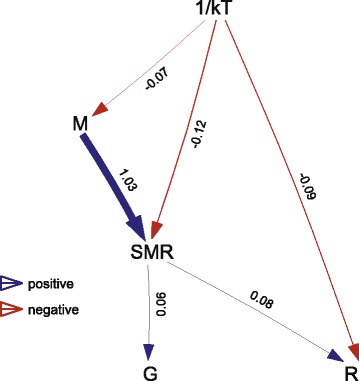
Visual representation of the averaged best-fitting path models (ΔCICc ≤2) for the squamate. Arrows indicate the direction of the path. See Fig. 3 for definitions for M, SMR, G, R and 1/kT

Phylogenetic confirmatory path analyses based on nine candidate path models (Fig. 3) show four directional associations: 1) between body mass and SMR; 2) between SMR and animal clade; 3) between SMR and reproductive mode; and 4) between temperature and body mass (Fig. 4; Tables 4 and 5). There are positive effects of body mass on SMR, of SMR on reproductive mode, of SMR on animal clade, and of temperature on body mass (Fig. 4; Tables 4 and 5).

**Table 4:** Results of the phylogenetic path analyses, ranking the candidate models according to their CICc values. The models with ΔCICc < 2 are in bold

Model	*k*	*q*	*C*	*P*	CICc	ΔCICc	*w*i
**five**	**4**	**11**	**2.983**	**0.935**	**26.417**	**0.000**	**0.225**
**eight**	**3**	**12**	**1.327**	**0.970**	**27.030**	**0.614**	**0.165**
**four**	**5**	**10**	**6.075**	**0.809**	**27.264**	**0.846**	**0.147**
**two**	**6**	**9**	**9.355**	**0.809**	**27.264**	**1.906**	**0.087**
seven	3	12	2.765	0.672	28.323	2.052	0.081
nine	3	12	2.810	0.832	28.515	2.098	0.079
three	5	10	7.391	0.688	28.580	2.162	0.076
one	6	9	8.676	0.730	28.644	2.227	0.074
six	4	11	4.402	0.819	28.837	2.419	0.067

*k* number of independence claims, *q* number of parameters, *C* Fisher’s *C* statistics, *CICc* C-statistic Information Criterion, ΔCICc difference in CICc from the best-fitting model, *w*i CICc weights

**Table 5:** Path statistics of the average and the best-fitting model from the phylogenetic path analyses in the squamates. For each model, the standardized regression coefficients are listed with their lower and upper 95% confidence limits. Coefficients with confidence intervals excluding 0 are in bold

Path	Top model	
M → SMR	**1.033**	**[0.976 / 1.090]**
SMR → G	**0.058**	**[0.052 / 0.064]**
SMR → R	**0.082**	**[0.043 / 0.121]**
1/kT → SMR	**−0.119**	**[−0.168 / –0.070]**
1/kT → R	−0.089	[−0.205 / 0.027]
1/kT → M	−0.07	[−0.182 / 0.042]

*M* body mass, *SMR* standard metabolic rate, *G* animal group (lizard vs snake), *R* reproductive mode (oviparous vs viviparous), *1/kT* 1/temperature

## Discussion

Our analyses show that: (1) snakes have lower SMRs (both tSMRs and mSMRs) than do lizards; (2) viviparous species have lower SMRs than do oviparous species; and (3) directional associations between SMR and animal clade or reproductive mode are evident. As elevated SMRs indicate increased costs of maintenance, our results are therefore consistent with the hypothesis that species or taxa with high maintenance costs would be less likely to invest more energy in reproduction or to evolve an energetically expensive mode of reproduction. We were unable to find sex-specific data, but our results still provide an important insight into evolved changes in maintenance costs for the following two reasons. First, although playing their respective sex-specific roles in reproduction, male and female reptiles share important similarities, including SMRs measured in the non-reproductive season. In all metabolic studies of reptiles where adequate data (allowing determination of whether differences in rate exist between sexes) are available, females generally have been found to have either as high or lower metabolic rates than males, with the rates often but not always elevated only when they are gravid [15, 29–31]. Second, reproduction is energetically expensive for females as well as for males and, unlike the endothermic amniotes (mammals and birds), many reptiles have the ability to decouple the time of feeding (energy acquisition) from reproduction (energy expenditure) and rely to a large degree on stored energy rather than currently acquired nutrients to fuel reproduction [10, 16]. Reduced maintenance costs may translate into an increased amount of energy channeled into growth (and thus the increased future reproductive potential), current reproduction, or both. In any case, the energetic savings of reduced maintenance costs will enhance reproduction.

Despite the fact that lizards and snakes are phylogenetically closely related, they differ in many important anatomical, ecological and behavioral characteristics [16, 24]. It is therefore not unexpected that they also differ physiologically. Importantly, however, our study shows for the first time that the evolutionary transition from lizard-like to snake-like body form alters maintenance metabolism of the organisms involved. We suggest three hypotheses to explain why snakes have evolved reduced maintenance costs. First, it is less energetically costly to spread body load evenly over the ventral surface as is the case for snakes, than to raise a body off the ground with limbs as is the case for most lizards [11]. Second, lizards and snakes have different ratios of surface area to body size (volume or mass) because they differ substantially in body shape. As a positive correlation between the body-mass allometry of metabolic rates and the allometry of body surface area also is present in ectotherms [32], lower maintenance costs may be associated with relatively smaller surface areas in relation to body size in snakes whose body plan is simpler than that of lizards. Third, the difference in relative clutch mass (RCM, a ratio of clutch mass to maternal mass) between lizards and snakes suggests that their responses to selection for this life-history trait differ substantially [12, 13, 16]. Given that there is a trade-off in the energy allocation between maintenance and reproduction, selection would favor the evolution of high RCM in snakes that reduced maintenance costs compared to lizards.

Our study also is the first to demonstrate in squamates that the evolutionary transition in reproductive mode (from oviparous to viviparous reproduction) alters maintenance metabolism. We suggest two explanations for why viviparous species have evolved reduced maintenance costs. First, there may be a high daily energetic cost involved in maintaining young within the body of the female [15]. Thus, even if maintenance costs for oviparous and viviparous species are equal on a daily basis, the longer gestation length of viviparous species still would result in higher energetic costs. Second, females of many viviparous species cease feeding in late stages of pregnancy [16], further increasing energetic constraints on this mode of reproduction. It is clear that, if reproduction is energetically constrained, then a reduction in maintenance costs among viviparous species would reduce this constraint.

## Conclusion

The transition from lizard-like to snake-like body form and the transition from oviparity to viviparity are major evolutionary transitions in vertebrates, which likely alter many aspects of biology and ecology of the organisms involved [33, 34]. Both transitions are common in squamate reptiles and have occurred repeatedly among closely related species, with fully legged and fully legless species sometimes occurring within the same genus and oviparous and viviparous individuals even within the same species [33, 34]. That maintenance metabolism relates to body plan (e.g. armored vs. unarmored species [35]) has been discussed in mammals, so has been the relationship between maintenance metabolism and reproductive mode (e.g., marsupial vs. placental species [5], and altricial vs. precocial species [36]). Our study is the first to demonstrate in squamates that evolutionary transitions in body plan (from lizard-like to snake-like body form) and reproductive mode (from oviparous to viviparous reproduction) alter maintenance metabolism.

## Additional files

Additional file 1: **Table S1.** Lizards (80 oviparous and 35 viviparous species) and snakes (48 oviparous and 33 viviparous species) for which data on standard metabolic rate (SMR, ml O_2_/h), body mass (g) and temperature (°C, at which SMRs were measured) have been available. Reprod mode: O: oviparous; V: viviparous. *: legless lizards. (DOC 481 kb)

Additional file 2: **Table S2.** Parameters of regressions (between tSMR and body mass and between mSMR and temperatures) estimated with ordinary least squares (OLS) and phylogenetic generalized least squares (PGLS) regression models. CI: confidence interval; ^**^ *P* <  0.0001; ^a^ the PGLS model is significantly better than the OLS model (likelihood ratio test). (DOC 45 kb)

Additional file 3: **Table S3.** ANOVA output of the PGLS model {ln(tSMR) ~ R × G × ln(M)} evaluating the effects of reproductive mode (R), animal clade (G) and body mass (M) on temperature-corrected standard metabolic rate (tSMR). (DOC 44 kb)

Additional file 4: **Table S4.** ANOVA output of the PGLS model {ln(mSMR) ~ R × G × T} evaluating the effects of reproductive mode (R), animal clade (G) and temperature (T) on mass-corrected standard metabolic rate (mSMR). (DOC 38 kb)

Additional file 5: **Table S5.** Results of the phylogenetic path analyses, ranking the candidate models according to their CICc values. The models with ΔCICc < 2 are in bold. (DOC 43 kb)

Additional file 6: **Table S6.** Path statistics of the average and the best-fitting model from the phylogenetic path analyses in the squamates. For each model, the standardized regression coefficients are listed with their lower and upper 95% confidence limits. Coefficients with confidence intervals excluding 0 are in bold. (DOC 34 kb)

## Abbreviations

AIC: Akaike’s Information Criterion
ANOVA: Analysis of variance
CICc: C-statistic Information Criterion
LRT: Likelihood-ratio test
mSMR: Mass-corrected standard metabolic rate
OLS: Ordinary least squares regression
PGLS: Phylogenetic generalized least squares regression
RCM: Relative clutch mass
SMR: Standard metabolic rate
tSMR: Temperature-corrected standard metabolic rate
