# Supplementary material for: Evolutionary transitions in body plan and reproductive mode alter maintenance metabolism in squamates
Source: BMC Evol Biol. 2018 Apr 3;18:45. doi: 10.1186/s12862-018-1166-5 (PMC5883405; doi:10.1186/s12862-018-1166-5)
Supplement: Supplementary file 1 — Table S1. Lizards (80 oviparous and 35 viviparous species) and snakes (48 oviparous and 33 viviparous species) for which data on standard metabolic rate (SMR, ml O2/h), body mass (g) and temperature (°C, at which SMRs were measured) have been available. Reprod mode: O: oviparous; V: viviparous. *: legless lizards. (DOC 481 kb) [file 12862_2018_1166_MOESM1_ESM.doc]

**Table S1.** Lizards (80 oviparous and 35 viviparous species) and snakes (48 oviparous and 33 viviparous species) for which data on standard metabolic rate (SMR, ml O_2_/h), body mass (g) and temperature (°C, at which SMRs were measured) have been available. Reprod mode: O: oviparous; V: viviparous. *: legless lizards

|  | Family | Species | Reprod mode | SMR | Body mass | Temperature | Reference |
| --- | --- | --- | --- | --- | --- | --- | --- |
| Lizards | | | | | | | |
| 1 | Agamidae | *Intellagama lesueurii* | O | 40.32 | 504 | 30 | [1,2] |
| 2 | Agamidae | *Pogona barbata* | O | 36.55 | 373 | 30 | [3] |
| 3 | Agamidae | *Pogona vitticeps* | O | 26.9 | 250 | 37 | [4] |
| 4 | Agamidae | *Phrynocephalus przewalskii* | O | 3.14 | 71.3 | 30 | [5] |
| 5 | Agamidae | *Uromastyx philbyi* | O | 8.36 | 117.73 | 30 | [6] |
| 6 | Anguidae | *Elgaria multicarinatus* | O | 4.93 | 29 | 30 | [7] |
| 7 | Crotaphytidae | *Crotaphytus collaris* | O | 5.4 | 30 | 30 | [8] |
| 8 | Dactyloidae | *Anolis acutus* | O | 1.25 | 4.3 | 30 | [9] |
| 9 | Dactyloidae | *Anolis bonairensis* | O | 1.7 | 12 | 33 | [10] |
| 10 | Dactyloidae | *Anolis carolinensis* | O | 0.85 | 4.5 | 30 | [11] |
| 11 | Dactyloidae | *Anolis limifrons* | O | 0.34 | 1.4 | 30 | [12] |
| 12 | Dactyloidae | *Anolis saagrei* | O | 1.105 | 2.8 | 30 | [13] |
| 13 | Gekkonidae | *Gekko gecko* | O | 5.93 | 61.5 | 30 | [14] |
| 14 | Gekkonidae | *Hemidactylus frenatus* | O | 0.92 | 4.4 | 30 | [15] |
| 15 | Gekkonidae | *Rhoptropus afer* | O | 0.173 | 2.29 | 30 | [16] |
| 16 | Iguanidae | *Amblyrhynchus cristatus* | O | 106.6 | 2600 | 25 | [17] |
| 17 | Iguanidae | *Dipsosaurus dorsalis* | O | 4.59 | 51 | 30 | [17] |
| 18 | Iguanidae | *Eurolophosaurus divaricatus* | O | 2.95 | 19.67 | 35 | [18] |
| 19 | Iguanidae | *Iguana iguana* | O | 64.39 | 795 | 30 | [19] |
| 20 | Iguanidae | *Sauromalus obesus* | O | 10.8 | 150 | 30 | [20] |
| 21 | Iguanidae | *Tropidurus hispidus* | O | 8.5 | 38.6 | 35 | [18] |
| 22 | Iguanidae | *Tropidurus hygomi* | O | 1.41 | 7.85 | 35 | [18] |
| 23 | Iguanidae | *Tropidurus insulanus* | O | 6.84 | 15.9 | 35 | [18] |
| 24 | Iguanidae | *Tropidurus itambers* | O | 7.47 | 21.34 | 35 | [18] |
| 25 | Iguanidae | *Tropidurus oreadicus* | O | 4.38 | 21.89 | 35 | [18] |
| 26 | Iguanidae | *Tropidurus psammonastes* | O | 5.67 | 25.78 | 35 | [18] |
| 27 | Iguanidae | *Tropidurus torquatus* | O | 5.83 | 27.76 | 35 | [18] |
| 28 | Lacertidae | *Acanthodactylus bosianus* | O | 1.95 | 7.8 | 30 | [21] |
| 29 | Lacertidae | *Acanthodactylus pardalis* | O | 3.88 | 9.7 | 30 | [21] |
| 30 | Lacertidae | *Acanthodactylus schreiberi* | O | 2.18 | 10.9 | 30 | [21] |
| 31 | Lacertidae | *Acanthodactylus scutellatus* | O | 1.25 | 6.6 | 30 | [21] |
| 32 | Lacertidae | *Teira dugesii* | O | 2.4 | 8 | 37 | [4] |
| 33 | Liolarmidae | *Liolaemus adaucan* | O | 0.57 | 5.51 | 30 | [22] |
| 34 | Liolarmidae | *Liolaemus calchaqui* | O | 0.49 | 5.78 | 30 | [22] |
| 35 | Liolarmidae | *Liolaemus chacoenis* | O | 0.16 | 1.21 | 30 | [22] |
| 36 | Liolarmidae | *Liolaemus cuyanus* | O | 1.7 | 14.83 | 30 | [22] |
| 37 | Liolarmidae | *Liolaemus darwinii* | O | 0.85 | 5.67 | 30 | [22] |
| 38 | Liolarmidae | *Liolaemus koslowskyi* | O | 1 | 6.68 | 30 | [22] |
| 39 | Liolarmidae | *Liolaemus laurenti* | O | 051 | 3.7 | 30 | [22] |
| 40 | Liolarmidae | *Liolaemus olongasta* | O | 0.86 | 4.51 | 30 | [22] |
| 41 | Liolarmidae | *Liolaemus quilmes* | O | 0.68 | 5.12 | 30 | [22] |
| 42 | Phrynosomatidae | *Petrosaurus mearnsi* | O | 2.38 | 14 | 30 | [23] |
| 43 | Phrynosomatidae | *Phrynosoma cornutum* | O | 5.81 | 35 | 35 | [24] |
| 44 | Phrynosomatidae | *Phrynosoma mcalli* | O | 2.72 | 16 | 30 | [25] |
| 45 | Phrynosomatidae | *Sceloporus graciosus* | O | 0.82 | 5 | 30 | [26] |
| 46 | Phrynosomatidae | *Sceloporus occidentalis* | O | 1.89 | 12.39 | 35 | [27] |
| 47 | Phrynosomatidae | *Sceloporus olivaceus* | O | 3.94 | 13 | 30 | [28] |
| 48 | Phrynosomatidae | *Sceloporus undulatus* | O | 2.01 | 4 | 30 | [29] |
| 49 | Phrynosomatidae | *Uta stansburiana* | O | 0.72 | 4.16 | 30 | [16] |
| 50 | Pygopodidae | *Lialis burtonis** | O | 0.58 | 15.2 | 30 | [30] |
| 51 | Pygopodidae | *Pygopus lepidopodus** | O | 1.77 | 29.5 | 30 | [30] |
| 52 | Scincidae | *Ctenotus labillardieri* | O | 0.2 | 2.8 | 20 | [31] |
| 53 | Scincidae | *Ctenotus lateralis* | O | 0.31 | 1 | 30 | [32] |
| 54 | Scincidae | *Plestiodon fasciatus* | O | 1.68 | 7 | 30 | [33] |
| 55 | Scincidae | *Plestiodon inexpectatus* | O | 1.47 | 9.6 | 30 | [14] |
| 56 | Scincidae | *Plestiodon laticeps* | O | 4.77 | 27.7 | 30 | [34] |
| 57 | Scincinae | *Plestiodon obsoletus* | O | 5.1 | 30 | 30 | [35] |
| 58 | Sphaerodactylidae | *Gonotodes antillensis* | O | 0.3 | 1.8 | 34 | [10] |
| 59 | Sphaerodactylidae | *Sphaerodactylus beattyi* | O | 0.9 | 0.4 | 30 | [36] |
| 60 | Sphaerodactylidae | *Sphaerodactylus* *macrolepis* | O | 0.13 | 0.5 | 30 | [37] |
| 61 | Teiidae | *Aspidoscelis burti* | O | 2.22 | 17.73 | 40 | [38] |
| 62 | Teiidae | *Cnemidophorus murinus* | O | 15.55 | 85 | 40 | [10] |
| 63 | Teiidae | *Cnemidophorus tigris* | O | 2.52 | 18 | 30 | [39] |
| 64 | Varanidae | *Varanus acanthurus* | O | 6.24 | 53.4 | 35 | [40] |
| 65 | Varanidae | *Varanus albigularis* | O | 149.26 | 963 | 35 | [41] |
| 66 | Varanidae | *Varanus brevicaudus* | O | 2.71 | 17.4 | 35 | [40] |
| 67 | Varanidae | *Varanus caudolineatus* | O | 2.26 | 13.1 | 35 | [40] |
| 68 | Varanidae | *Varanus eremius* | O | 6.16 | 35.9 | 35 | [40] |
| 69 | Varanidae | *Varanus giganteus* | O | 237 | 2502 | 35 | [40] |
| 70 | Varanidae | *Varanus gilleni* | O | 3.58 | 20.0 | 35 | [40] |
| 71 | Varanidae | *Varanus glauerti* | O | 12.6 | 33.8 | 35 | [42] |
| 72 | Varanidae | *Varanus gouldii* | O | 38.1 | 303 | 35 | [42] |
| 73 | Varanidae | *Varanus kingorum* | O | 3.37 | 19.8 | 35 | [42] |
| 74 | Varanidae | *Varanus mertensi* | O | 72 | 904 | 35 | [42] |
| 75 | Varanidae | *Varanus mitchelli* | O | 32.9 | 184 | 35 | [42] |
| 76 | Varanidae | *Varanus panoptes* | O | 179 | 1622 | 35 | [40] |
| 77 | Varanidae | *Varanus rosenbergi* | O | 284 | 2071 | 35 | [40] |
| 78 | Varanidae | *Varanus scalaris* | O | 19.33 | 88.33 | 35 | [42] |
| 79 | Varanidae | *Varanus storri* | O | 5.65 | 27.63 | 35 | [42] |
| 80 | Varanidae | *Varanus tristis* | O | 15.7 | 99 | 35 | [40] |
| 81 | Anguidae | *Anguis fragilis** | V | 0.77 | 11.2 | 30 | [14] |
| 82 | Anniellidae | *Anniella pulchra** | V | 0.34 | 4.5 | 20 | [43] |
| 83 | Cordylidae | *Pseudocordylus melanotus* | V | 4.82 | 44.7 | 30 | [44] |
| 84 | Diplodactylidae | *Naultinus manukanus* | V | 0.67 | 6 | 26 | [45] |
| 85 | Diplodactylidae | *Toropuku stephensi* | V | 0.91 | 8.2 | 26 | [45] |
| 86 | Diplodactylidae | *Woodworthia chrysosirsticus* | V | 0.61 | 5.9 | 26 | [45] |
| 87 | Diplodactylidae | *Woodworthia maculatus* | V | 0.77 | 7.8 | 26 | [45] |
| 88 | Liolaemidae | *Liolaemus albiceps* | V | 0.88 | 12.08 | 30 | [22] |
| 89 | Liolaemidae | *Liolaemus bellii* | V | 1.67 | 12.1 | 30 | [22] |
| 90 | Liolaemidae | *Liolaemus crepuscularis* | V | 0.32 | 2.11 | 30 | [22] |
| 91 | Liolaemidae | *Liolaemus espinozai* | V | 0.5 | 4.85 | 30 | [22] |
| 92 | Liolaemidae | *Liolaemus irregularis* | V | 1.56 | 15.88 | 30 | [22] |
| 93 | Liolaemidae | *Liolaemus lavillai* | V | 0.31 | 3.79 | 30 | [22] |
| 94 | Liolaemidae | *Liolaemus ornatus* | V | 0.79 | 4.07 | 30 | [22] |
| 95 | Liolaemidae | *Phymaturus dorsimaculatus* | V | 3.51 | 43.39 | 30 | [22] |
| 96 | Liolaemidae | *Phymaturus exelsus* | V | 1.19 | 20.53 | 30 | [22] |
| 97 | Liolaemidae | *Phymaturus spectabilis* | V | 1.25 | 15.81 | 30 | [22] |
| 98 | Liolaemidae | *Phymaturus tenebrosus* | V | 2.85 | 24.85 | 30 | [22] |
| 99 | Phrynosomatidae | *Phrynosoma douglassi* | V | 4.78 | 28 | 35 | [25] |
| 100 | Scincidae | *Acontias meleagris** | V | 0.68 | 7.3 | 33 | [46] |
| 101 | Scincidae | *Chalcides ocellatus* | V | 2.35 | 25 | 33 | [47] |
| 102 | Scincidae | *Chalcides sepsoides* | V | 0.68 | 7.4 | 30 | [14] |
| 103 | Scincidae | *Egernia cunninghami* | V | 22.71 | 261 | 30 | [1,2] |
| 104 | Scincidae | *Oligosoma aeneum* | V | 0.37 | 2.6 | 26 | [45] |
| 105 | Scincidae | *Oligosoma macgregori* | V | 1.22 | 19 | 26 | [45] |
| 106 | Scincidae | *Oligosoma polychrome* | V | 0.53 | 3.3 | 26 | [45] |
| 107 | Scincidae | *Oligosoma zelandicum* | V | 0.56 | 3.8 | 26 | [45] |
| 108 | Scincidae | *Scelotes gronovii** | V | 0.25 | 1.1 | 33 | [46] |
| 109 | Scincidae | *Tiliqua rugosa* | V | 42.41 | 461 | 30 | [1,2] |
| 110 | Scincidae | *Tiliqua scincoides* | V | 44.37 | 493 | 30 | [48] |
| 111 | Xantusiidae | *Lepidophyma gaigeae* | V | 0.69 | 5 | 30 | [49] |
| 112 | Xantusiidae | *Lepidophyma smithi* | V | 1.75 | 25 | 30 | [49] |
| 113 | Xantusiidae | *Xantusia henshawi* | V | 0.54 | 3.5 | 30 | [49] |
| 114 | Xantusiidae | *Xantusia riversiana* | V | 1.99 | 19 | 30 | [49] |
| 115 | Xantusiidae | *Xantusia vigilis* | V | 0.31 | 1.5 | 30 | [49] |
| Snakes | | | | | | | |
| 116 | Colubridae | *Boaedon fuliginosus* | O | 0.68 | 16.7 | 25 | [50] |
| 117 | Colubridae | *Boiga irregularis* | O | 5.4 | 133 | 30 | [51] |
| 118 | Colubridae | *Chironius quadricarinatus* | O | 2.07 | 61 | 20 | [52] |
| 119 | Colubridae | *Coluber constrictor* | O | 21.9 | 223 | 30 | [53] |
| 120 | Colubridae | *Coluber flagellum* | O | 5.5 | 124 | 30 | [54] |
| 121 | Colubridae | *Dasypeltis inornata* | O | 3.14 | 71.3 | 30 | [55] |
| 122 | Colubridae | *Dasypeltis scabra* | O | 1.69 | 59.1 | 30 | [55] |
| 123 | Colubridae | *Diadophis punctatus* | O | 0.58 | 4.4 | 30 | [56] |
| 124 | Colubridae | *Dipsas albifrons* | O | 0.81 | 22 | 20 | [52] |
| 125 | Colubridae | *Erythrolamprus poecilogyrus* | O | 1.68 | 42 | 20 | [52] |
| 126 | Colubridae | *Hemorrhois ravergieri* | O | 7.9 | 136 | 30 | [57] |
| 127 | Colubridae | *Hydrodynastes gigas* | O | 85.76 | 2680 | 20 | [52] |
| 128 | Colubridae | *Lampropeltis getula* | O | 11.8 | 188 | 30 | [53] |
| 129 | Colubridae | *Liophis miliaris* | O | 31.28 | 401 | 30 | [58] |
| 130 | Colubridae | *Mastigodryas bifossatus* | O | 31.6 | 735 | 20 | [52] |
| 131 | Colubridae | *Natrix maura* | O | 2 | 25 | 30 | [59] |
| 132 | Colubridae | *Natrix natrix* | O | 7.56 | 84 | 20 | [60] |
| 133 | Colubridae | *Natrix natrix helvetica* | O | 13 | 100 | 30 | [59] |
| 134 | Colubridae | *Natrix natrix persa* | O | 2.9 | 40 | 30 | [59] |
| 135 | Colubridae | *Oxyrhopus trigeminus* | O | 3.43 | 98 | 20 | [52] |
| 136 | Colubridae | *Pantherophis guttatus* | O | 30.4 | 800 | 25 | [61] |
| 137 | Colubridae | *Philodryas olfersii* | O | 6.86 | 176 | 20 | [52] |
| 138 | Colubridae | *Philodryas patagoniensis* | O | 19.79 | 388 | 20 | [52] |
| 139 | Colubridae | *Pitcophis melanoleucus* | O | 38.56 | 548 | 30 | [62, 63] |
| 140 | Colubridae | *Rhagerhis moilensis* | O | 11.5 | 145 | 30 | [64] |
| 141 | Colubridae | *Salvadora hexalepis* | O | 2.99 | 65 | 30 | [65] |
| 142 | Colubridae | *Sibynomorphus mikanii* | O | 1.24 | 11 | 20 | [57] |
| 143 | Colubridae | *Sphalerosophis cliffordii* | O | 47.74 | 351 | 30 | [66] |
| 144 | Colubridae | *Spalerosophis diadema* | O | 12.7 | 218 | 30 | [57] |
| 145 | Colubridae | *Thamnodynastes strigatus* | O | 2.25 | 55 | 20 | [52] |
| 146 | Colubridae | *Tropidodryas serra* | O | 4.72 | 135 | 20 | [52] |
| 147 | Colubridae | *Xenodon guentheri* | O | 1.75 | 50 | 20 | [52] |
| 148 | Colubridae | *Xenodon merremii* | O | 21.59 | 502 | 20 | [52] |
| 149 | Colubridae | *Xenodon neuwiedii* | O | 1.96 | 53 | 20 | [52] |
| 150 | Elapidae | *Pseudonaja nuchalis* | O | 14.4 | 214 | 30 | [67] |
| 151 | Pythonidae | *Antaresia childreni* | O | 21.9 | 332 | 30 | [67] |
| 152 | Pythonidae | *Antaresia stimsoni* | O | 20.2 | 350 | 30 | [67] |
| 153 | Pythonidae | *Aspidites melanocephalus* | O | 89.52 | 1028 | 30 | [67] |
| 154 | Pythonidae | *Liasis fuscus* | O | 35.88 | 1307 | 30 | [67] |
| 155 | Pythonidae | *Liasis olivaceus* | O | 119.4 | 3323 | 30 | [67] |
| 156 | Pythonidae | *Malayopython reticulatus* | O | 334.5 | 14325 | 30 | [68] |
| 157 | Pythonidae | *Morelia spilota* | O | 37.9 | 1050 | 30 | [68] |
| 158 | Pythonidae | *Python curtis* | O | 45.8 | 2375 | 30 | [68] |
| 159 | Pythonidae | *Python molurus* | O | 222.66 | 12370 | 30 | [69,70] |
| 160 | Pythonidae | *Python regius* | O | 31.9 | 1523 | 30 | [71] |
| 161 | Pythonidae | *Python sebae* | O | 286.8 | 16150 | 30 | [68] |
| 162 | Viperidae | *Cerastes cerastes* | O | 8.6 | 121 | 30 | [64] |
| 163 | Viperidae | *Macrovipera palaestinae* | O | 24.4 | 581 | 30 | [57] |
| 164 | Acrochordiae | *Acrochordus arafurae* | V | 44.52 | 1048 | 30 | [67] |
| 165 | Boidae | *Acrantophis dumerili* | V | 48.2 | 2550 | 30 | [68] |
| 166 | Boidae | *Boa constrictor* | V | 126.1 | 7825 | 30 | [52] |
| 167 | Boidae | *Candoia carinatus* | V | 50.5 | 525 | 30 | [68] |
| 168 | Boidae | *Corallus caninus* | V | 14.6 | 550 | 30 | [68] |
| 169 | Boidae | *Corallus hortulanus* | V | 18.8 | 800 | 30 | [68] |
| 170 | Boidae | *Epicrates cenchria* | V | 12.3 | 425 | 30 | [68] |
| 171 | Boidae | *Eryx colubrinus* | V | 3 | 75 | 30 | [68] |
| 172 | Boidae | *Eunectes murinus* | V | 23.73 | 1130 | 20 | [52] |
| 173 | Boidae | *Eunectes notaeus* | V | 216 | 14400 | 20 | [52] |
| 174 | Boidae | *Lichanura roseofusca* | V | 21.98 | 314 | 30 | [72] |
| 175 | Boidae | *Lichanura trivirgata* | V | 5.9 | 175 | 30 | [68] |
| 176 | Colubridae | *Helicops modestus* | V | 10.78 | 196 | 30 | [58] |
| 177 | Colubridae | *Nerodia fasciata* | V | 3.69 | 50.8 | 25 | [73] |
| 178 | Colubridae | *Nerodia rhombifer* | V | 22.61 | 238 | 30 | [74] |
| 179 | Colubridae | *Nerodia sipedon* | V | 43.84 | 292 | 30 | [75] |
| 180 | Colubridae | *Nerodia taxispilota* | V | 42.85 | 372 | 30 | [75] |
| 181 | Colubridae | *Storeria dekayi* | V | 0.63 | 7.2 | 30 | [76] |
| 182 | Colubridae | *Thamnophis proximus* | V | 4.65 | 31 | 30 | [65] |
| 183 | Colubridae | *Thamnophis sirtalis* | V | 6.6 | 200 | 29 | [77,78] |
| 184 | Elapidae | *Acanthophis praelongus* | V | 4.74 | 106 | 30 | [67] |
| 185 | Viperidae | *Agkistrodon piscivorus* | V | 5.2 | 250 | 30 | [79] |
| 186 | Viperidae | *Bothrops moojeni* | V | 84.7 | 410 | 30 | [80] |
| 187 | Viperidae | *Crotalus adamanteus* | V | 102.8 | 3373 | 30 | [81] |
| 188 | Viperidae | *Crotalus atrox* | V | 9.1 | 300 | 30 | [82] |
| 189 | Viperidae | *Crotalus cerastes* | V | 4 | 129 | 30 | [54] |
| 190 | Viperidae | *Crotalus durrisus* | V | 2.2 | 50 | 30 | [83] |
| 191 | Viperidae | *Crotalus horridus* | V | 6.1 | 300 | 30 | [84] |
| 192 | Viperidae | *Crotalus lepidus* | V | 2.5 | 115 | 30 | [85] |
| 193 | Viperidae | *Crotalus molossus* | V | 4.5 | 300 | 30 | [85] |
| 194 | Viperidae | *Crotalus viridis* | V | 24.1 | 301 | 30 | [70] |
| 195 | Viperidae | *Vipera aspis* | V | 4.3 | 85 | 32 | [86] |
| 196 | Viperidae | *Vipera berus* | V | 9.5 | 70 | 30 | [64] |

**References**

1. Wilson KJ. The relationship of oxygen supply for activity to body temperature in four species of lizards. Copeia. 1974;1974;920–34.
2. Wilson KJ. The relationship of activity, energy, metabolism, and body temperature in four species of lizards. Ph.D. Thesis, Monash University; 1971.
3. Bartholomew GA, Tucker VA. Size, body temperature, thermal conductance, oxygen consumption, and heart rate in Australian varanid lizards. Physiol Zool. 1963;37:341–54.
4. Brookes PS, Buckingham JA, Tenreiro AM, Hulbert A, Brand MD. The proton permeability of the inner membrane of liver mitochondria from ectothermic and endothermic vertebrates and from obese rats: correlations with standard metabolic rate and phospholipid fatty acid composition. Comp Biochem Physiol B. 1998;119:325–34.
5. He JZ, Xiu MH, Tang XL, Wang NB, Xin Y, Li WX, Chen Q. Thermoregulatory and metabolic responses to hypoxia in the oviparous lizard, *Phrynocephalus przewalskii*. Comp Biochenm Physiol A. 2013;165:207–13.
6. 6.Zari TA. Seasonal metabolic acclimatization in the herbivorous desert lizard *Uromastyx philbyi* (Reptilia: Agamidea) from western Saudi Arabia. J Therml Biol. 2016;60:180–5.
7. Dawson WR, Templeton JR. Physiological responses to temperature in the alligator lizard, *Gerrhonotus multicarinatus*. Ecology. 1966;47:759–65.
8. Dawson WR, Templeton JR. Physiological responses to temperature in the lizard *Crotaphytus collaris*. Physiol Zool. 1963;36:219–36.
9. McManus JJ, Nellis DW. Temperature and metabolism of a tropical lizard, *Anolis acutus*. Comp Biochem Physiol A. 1973;45:403–10.
10. Bennett AF, Gorman GC. 1979 Population density and energetics of lizards on a tropical island. Oecologia. 1979;42:339–58.
11. Maher MJ, Levendahl BH. The effect of the thyroid gland on the oxidative metabolism of the lizard, *Anolis carolinensis*. J Exp Zool. 1959;140:169–89.
12. Andrews RM, Asato T. Energy utilization of a tropical lizard. Comp Biochem Physiol A*.* 1977;58:57–62.
13. Steffen JE, Appel AG. The effect of temperature on standrad metabolic rate of Brown anoles. Amphibia-Reptilia. 2012;33:297–302.
14. Andrews RM, Pough FH. Metabolism of squamate reptiles: allometric and ecological relationship. Physiol Zool. 1985;58:214–31.
15. Suyder GK, Weathers WW. Physiological responses to temperature in the tropical lizard, *Hemidactylus frenatus* (Sauria: Gekkonidae). Herpetologica. 1976;32:252–6.
16. Peterson CC. Paradoxically low metabolic rate of the diurnal gecko *Rhoptropus afer*. Copeia, 1990;1990:233–7.
17. Moberly, W.R. 1963. Hibernation in the desert iguana, *Dipsosaurus dorsalis*. Physiol Zool. 1963;36:152–60.
18. Kohlsdorf T, Navas C. Evolutiona of form and function: morphophysiological relationships and locomotor performance in tropidurine lizards. J Zool. 2012;288:41–9.
19. Moberly WR. The physiological correlates of activity in the Common Iguana, *Iguana iguana*. Ph.D. Dissertation, University of Michigan, Ann Arbor; 1966.
20. 20.Boyer DR. Interaction of temperature and hypoxia on respiratory and cardiac responses in the lizards, *Sauromalus obesus*. Comp Biochem Physiol. 1967;20:437–47.
21. Duvdevani I, Borut A. Oxygen consumption and evaporative water loss in four species of *Acanthodactylus* (Lacertidae). Copeia. 1974;1974:155–64.
22. Cruz FB, Antenucci D, Luna F, Abdala CS, Vega LF. Energetics in Liolaemini lizards: implications of a small body size and ecological conservatism. J Comp Physiol B. 2011;181:373–82.
23. Murrish DE, Vance VJ. Physiological responses to temperature acclimation in the lizard *Uta mearnsi*. Comp Biochem Physiol. 1968;27:329–37.
24. Prieto AA, Whitford WG. Physiological responses to temperature in the horned lizards, *Phrynosoma cornutum* and *Phrynosoma douglassi*. Copeia. 1971;1971:498–504.
25. Mayhew WW. Hibernation in the horned lizard, *Phrtnosoma mcalli*. Comp Biochem Physiol. 1965;16:103–19.
26. Mueller CF. Temperature and energy characteristics of the sagebrush lizard (*Sceloporus graciosus*) in Yellowstone National Park. Copeia*.* 1969;1969:153–60.
27. Tsuji JS. Seasonal profiles of standard metabolic rate of lizards (*Sceloporus occidentalis*) in relation to latitude. Physiol Zool. 1988;61:230-40.
28. Dutton RH, Fitzpatrick LC, Hughes JL. Energetics of the rusty lizard *Sceloporus olivaceus*. Ecology. 1975;56:1378–87.
29. Hughes JL, Fitzgerald LC, Ferguson GW, Beitinger TL. Oxygen consumption and temperature acclimation in the northern prairie swift *Sceloporus undulates garmani* from Kansas. Comp Biochem Physiol A. 1982;71:611–3.
30. Wall M, Thompson MB, Shine R. Does foraging mode affect metabolic responses to feeding? A study of pygopodid lizards. Curr Zool. 2013;59:618–25.
31. Dawson WR, Shoemaker VH, Licht P. Evaporative water losses of some small Australian lizards. Ecology. 1966;47:589–94.
32. Hudson JW, Bertram FW. Physiological responses to temperature in the ground skink, *Lygosoma laterale*. Physiol Zool. 1966;39:21–9.
33. Maher MJ. The role of the thyroid gland in the oxygen consumption of lizards. Gen Comp Endocrinol. 1965;5:320–5.
34. Watson CM, Burggren WW. Interspecific differences in metabolic rate and metabolic temperature sensitivity create distinct thermal ecological niches in lizards (*Plestiodon*). PLoS One. 2016;11:e0164713.
35. Dawson TJ, Hulbert AJ. Standard metabolism, body temperature, and surface areas of Australian marsupials. Am J Physiol. 1970;218:1233–8.
36. Snyder GK. Water loss and oxygen consumption in tropical *Sphaerodactylus*. Oecologia. 1979;38:107–10.
37. Snyder GK. Respiratory metabolism and evaporative water loss in a small tropical lizard. J Comp Physiol. 1975;104:13–8.
38. Cullum AJ. Comparisons of physiological performance in sexual and asexual Whiptail lizards (genus *Cnemidophorus*): implications for the role of heterozygosity. Am Nat. 1997;150:24–47.
39. Asplund KK. Metabolic scope and body temperatures of whiptail lizards (*Cnemidophorus*). Herpetologica. 1970;26:403–11.
40. Thompson GG, Withers PC. Standard and maximal metabolic rates of Goannas (Squamata: Varanidae). Physiol Zool. 1997;70:307–32.
41. Louw G, Young BA, Bligh J. Effect of thyroxine and noradrenaline on thermoregulation, cardiac rate and oxygen consumption in the monitor lizard *Varanus albigularis albigularis*. J Therm Biol. 1976;1:189–93.
42. Clemente CJ, Withers PC, Thompson GG. Metabolic rate and endurance capacity in Australian varanid lizards (Squamata: Varanidae: *Varanus*). Biol J Linn Soc. 2009;97:664–76.
43. Fusari M. Temperature responses pf standard aerobic metabolism by the California legless lizard, *Anniella pulchra*. Comp Biochem Physiol A. 1984;77:97–102.
44. McConnachie S. The effects of temperature on oxygen consumption in the lizard *Pseudocordylus melanotus* from Suikerbosrand Nature Reserve. Afr J Herpeol. 2014;63:57–69.
45. Hare KM, Pledger S, Thompson MB, Miller JH, Daugherty CH. Nocturnal lizards from a cool-temperate environment have high metabolic rates at low temperature. J Comp Physiol B. 2010;180:1173–82.
46. Withers PC. Physiological correlates of limblessness and fossoriality in scincid lizards. Copeia. 1981;1981:197–204.
47. Pough FH, Andrews RM. Individual and sibling–group variation in metabolism of lizards: implications for the origin of endothermy. Comp Biochem Physiol A. 1984;79:415–9.
48. Bartholomew GA, Tucker VA, Lee AK. Oxygen consumption, thermal conductance, and heart rate in the Australian skink, *Tiliqua scincoides*. Copeia. 1965;1965:169–73.
49. Mautz WJ. The metabolism of reclusive lizards, the Xantusiidae. Copeia. 1979;1979:577–84.
50. Roe JH, Hopkins WA, Snodgrass JW, Congdon JD. The influence of circadian rhythms on pre- and post-prandial metabolism in the snake *Lamprophis fuliginosus*. Comp Biochenm Physiol. 2004;139:159-68.
51. Anderson NL, Hetherington TE, Williams JB. Validation of the doubly labeled water method under low and high humidity to estimate metabolic rate and water flux in a tropical snake (*Boiga irregularis*). J Appl Physiol. 2003;95:184–91.
52. Galvão PE, Tarasantchi J, Guertzenstein P. Heat production of tropical snakes in relation to body weight and body surface. Am J Physiol. 1965;209:501–6.
53. Secor SM, Diamond JM. Evolution of regulatory responses in feeding in snakes. Physiol Biochem Zool. 2000;73:123–41.
54. Secor SM, Nagy KA. Bioenergetic correlates of foraging mode for the snakes *Crotalus cerastes* and *Masticophis flagellum*. Ecology. 1994;75:1600–14.
55. Greene S, McConnachie S, Secor S, Perrin M. The effects of body temperature and mass on the postprandial metabolic responses of the African egg-eating snakes *Dasypeltis scabra* and *Dasypeltis inornta*. Comp Biochem Physiol A. 2013;165:97–105.
56. Buikema AL Jr., Armitage KB. The effect of temperature on the metabolism of the prairie ringneck snake, *Diadophis punctatus arnyi* Kennicott. Herpetologica. 1969;25:194–206.
57. Dmi'el R. Effect of activity and temperature on metabolism and water loss in snakes. Am J Physiol. 1972;223:510–6.
58. Abe AS, Mendes EG. Effect of body size and temperature on oxygen uptake in the water snakes *Helicops modestus* and *Liphis miliaris* (Colubridae). Comp Biochem Physiol A. 1980;65:367–70.
59. Hailey A, Davies PMC. Lifestyle, latitude and activity metabolism of natricine snakes. J Zool. 1986;209:461–76.
60. Hill AV. The total energy exchanges of intact cold-blooded animals at rest. J Physiol. 1911;43:379–94.
61. Smith GC. Ecological energetics of three species of ectothermic vertebrates. Ecology. 1976;57:252–64.
62. Baldwin FM. Oxygen consumption at 20° in certain snakes *Pituophis sayi* and *Lampropeltis getulus holbrooki* with some notes on size and seasonal difference. Proc Iowa Acad Sci. 1928;35:313–8.
63. Greenwald OE. The effect of body temperature on oxygen consumption and heart rate in the Sonora gopher snake, *Pituophis catenifer affinis* Hallowell. Copeia. 1971;1971:98–106.
64. Al-Sadoon MD. 1991 Metabolic rate-temperature curves of the horned viper, *Cerastes cerastes gasperetti*, the Moila snake, *Malpolon moilensis*, and the adder *Vipera berus*. Comp Biochem Physiol A. 1991;99:119–22.
65. Jacobson ER, Whitford WG. Physiological responses to temperature in the patch–nosed snake, *Salvdora hexalepis*. Herpetologica. 1971;27:289–95.
66. Dmi’el R, Borut A. Thermal behavior, heat exchange, and metabolism in the desert snake *Spalerosophis cliffordi*. Physiol Zool. 1972;45:78–94.
67. Bedford GS, Christian KA. Standard metabolic rate and preferred body temperature in some Australian pythons. Aust J Zool. 1998;46:317–28.
68. Chappell MA, Ellis TM. Resting metabolic rates in boid snakes: allometric relationships and temperature effects. J Comp Physiol B. 1987;157:227–35.
69. Hutchison VH, Dowling HG, Vinegar A. Thermoregulation in a brooding female Indian python, *Python molurus bivittatus*. Science. 1966;151:694–6.
70. Vinegar A. Metabolisn, energetics, and thermoregulation during brooding of snakes of the genus *Python* (Reptilia, Boidae). Master Thesis, University of Rhode Island; 1968.
71. Ellis TM, Chappel MA. Metabolism, temperature relations, maternal behavior, and reproductive energetics in the ball python (*Python regius*). J Comp Physiol B. 1987;157:393–402.
72. Ruben JA. Aerobic and anaerobic metabolism during activity in snakes. J Comp Physiol B. 1976;109:147–57.
73. Hopkins WA, Rower CL, Congdon JD. Elevated trace element concentrations and standard metabolic rate in banded water snakes (*Nerodia fasciata*) exposed to coal combustion wastes. Environ Toxicol Chem. 1999;18:1258–63.
74. Jacobson ER, Whitford WG. The effect of acclimation on physiological responses to temperature in the snakes *Thamnophis proximus* and *Natrix rhombifera*. Comp Biochem Physiol. 1970;35:439–49.
75. Blem CR, Blem KL. Metabolic acclimation in three species of sympatric, semi–aquatic snakes. Comp Biochem Physiol A. 1990;97:259–64.
76. Clausen HJ. The effect of aggregation on the respiratory metabolism of the brown snake *Storeria dekayi*. J Cell Comp Physiol. 1936;8:367–86.
77. Aleksiuk M. Temperature-dependent shifts in the metabolism of a cool temperate reptile, *Thamnophis sirtalis parietalis*. Comp Biochem Physiol A. 1971;39:495–503.
78. Taylor BM, Davies PMC. Changes in the weight dependence of metabolism during the sloughing cycle of the snake *Thamnophis sirtalis parietalis*. Comp Biochem Physiol A. 1981;69:113–9.
79. Zaidan F, III. Geographic physiological variation and northern range limits in the cottonmouth (*Agkistrodon piscivorus leucostoma*). Ph.D. Thesis, University of Arkansas; 2002.
80. Cruz-Neto AP, Abe AS. Ontogenetic variation of oxygen uptake in the pitviper *Bothrops moojeni* (Serpentes, Viperidae). Comp Biochem Physiol A. 1994;108:549–54.
81. Dorcas ME, Hopkins WA, Roe JH. Effects of body mass and temperature on standard metabolic rate in the eastern diamondback rattlesnake (*Crotalus adamanteus*). Copeia. 2004;2004:145–51.
82. Beaupre SJ, Montgomery CE. The meaning and consequences of foraging mode in snakes. In: Reilly SM, McBrayer LD, Miles DB, editors. Lizard ecology: the evolutionary consequences of foraging mode. Cambridge: Cambridge University Press; 2007.
83. Cruz-Neto AP, Andrade DV, Abe AS. Energetic cost of predation: aerobic metabolism during prey ingestion by juvenile rattlesnakes, *Crotalus durissus*. J Herpetol. 1999;33:229–34.
84. Beaupre SJ, Zaidan FIII. Scaling of CO_2_ production of the timber rattlesnake (*Crotalus atrox*), with comments on cost of growth in neonates and comparative patterns. Physiol Biochem Zool. 2001;74:757–68.
85. Beaupre SJ. An ecological study of oxygen consumption in the mottled rock rattlesnake, *Crotalus lepidus lepidus*, and the black-tailed rattlesnake, *Crotalus molossus molossus*, from two populations. Physiol Zool. 1993;66:437–54.
86. Ladyman M, Bonnet X, Lourdais O, Bradshaw D, Naulleau G. Gestation, thermoregulation, and metabolism in a viviparous snake, *Vipera aspis*: evidence for fecundity-independent costs. Physiol Biochem Zool. 2003;76:497–510.
