# Supplementary material for: Evolutionary transitions in body plan and reproductive mode alter maintenance metabolism in squamates
Source: BMC Evol Biol. 2018 Apr 3;18:45. doi: 10.1186/s12862-018-1166-5 (PMC5883405; doi:10.1186/s12862-018-1166-5)
Supplement: Supplementary file 2 — Table S2. Parameters of regressions (between tSMR and body mass and between mSMR and temperatures) estimated with ordinary least squares (OLS) and phylogenetic generalized least squares (PGLS) regression models. CI: confidence interval; ** P < 0.0001; a the PGLS model is significantly better than the OLS model (likelihood ratio test). (DOC 45 kb) [file 12862_2018_1166_MOESM2_ESM.doc]

**Table S2** Parameters of regressions (between tSMR and body mass and between mSMR and temperatures) estimated with ordinary least squares (OLS) and phylogenetic generalized least squares (PGLS) regression models. CI: confidence interval; ^**^ *P* < 0.0001; ^a^ the PGLS model is significantly better than the OLS model (likelihood ratio test)

| Model | *N* | Slope (±SE) | *r*^2^ | λ (95%CI) | AIC | ln likelihood | *F*_1, 169_ | *P-*value |
| --- | --- | --- | --- | --- | --- | --- | --- | --- |
| tSMR vs body mass | | | | | | | | |
| OLS | 171 | 0.76 (0.02)^**^ | 0.88 |  | 333.9 | −162.0 | 1258.12 | <0.001 |
| PGLS | 171 | 0.76 (0.02)^**^ | 0.88 | 0 | 331.9 | −160.0 | 1258.2 | <0.001 |
|  | 171 | 0.86 (0.03)^**^ | 0.87 | 0.82 (0.58/0.93) | 251.6 | −119.8^a^ | 1119.70 | <0.001 |
|  | 171 | 0.84 (0.03)^**^ | 0.81 | 1 | 329.0 | −158.5 | 736.66 | <0.001 |
| mSMR vs temperature | | | | | | | | |
| OLS | 171 | −0.67 (0.09)^**^ | 0.25 |  | 320.6 | −155.3 | 55.81 | <0.001 |
| PGLS | 171 | −0.67 (0.09)^**^ | 0.25 | 0 | 318.6 | −153.3 | 55.79 | <0.001 |
|  | 171 | −0.36 (0.10)^**^ | 0.17 | 0.80 (0.52 / 0.93) | 252.2 | −120.1^a^ | 14.06 | <0.001 |
|  | 171 | −0.29 (0.10)^**^ | 0.14 | 1 | 325.5 | −156.8 | 8.83 | <0.01 |
