# Supplementary material for: Evolutionary transitions in body plan and reproductive mode alter maintenance metabolism in squamates
Source: BMC Evol Biol. 2018 Apr 3;18:45. doi: 10.1186/s12862-018-1166-5 (PMC5883405; doi:10.1186/s12862-018-1166-5)
Supplement: Supplementary file 3 — Table S3. ANOVA output of the PGLS model {ln(tSMR) ~ R × G × ln(M)} evaluating the effects of reproductive mode (R), animal clade (G) and body mass (M) on temperature-corrected standard metabolic rate (tSMR). (DOC 44 kb) [file 12862_2018_1166_MOESM3_ESM.doc]

**Table S3** ANOVA output of the PGLS model {ln(tSMR) ~ R × G × ln(M)} evaluating the effects of reproductive mode (R), animal clade (G) and body mass (M) on temperature-corrected standard metabolic rate (tSMR)

| Source | *df* | SQ | MSQ | *F* | *P*-value |
| --- | --- | --- | --- | --- | --- |
| Reproductive mode | 1 | 0.012 | 0.012 | 4.26 | 0.041 |
| Animal clade | 1 | 0.009 | 0.009 | 4.43 | 0.037 |
| Body mass | 1 | 3.329 | 3.329 | 1216.12 | < 0.001 |
| R × G | 1 | 0.014 | 0.014 | 5.03 | 0.026 |
| R × M | 1 | 0.001 | 0.001 | 0.42 | 0.518 |
| G × M | 1 | <0.001 | <0.001 | 0.13 | 0.721 |
| R × G × M | 1 | <0.001 | <0.001 | 0.21 | 0.648 |
| Residuals | 163 | 0.446 | 0.003 |  |  |

**Table S6** Path statistics of the average and the best-fitting model from the phylogenetic path analyses in the squamates. For each model, the standardized regression coefficients are listed with their lower and upper 95% confidence limits. Coefficients with confidence intervals excluding 0 are highlighted in bold.

| Path | Top model | |
| --- | --- | --- |
| M → SMR | **1.241** | **[0.653 / 1.829]** |
| SMR → G | **0.586** | **[0.429 / 0.743]** |
| SMR → R | **0.944** | **[0.866 / 1.022]** |
| 1/kT → SMR | **‒0.681** | **[‒0.857 / ‒0.505]** |
| 1/kT → R | ‒0135 | [‒0.331 / 0.061] |
| 1/kT → M | ‒0.196 | [‒0.412 / 0.020] |

M = body mass; SMR = standard metabolic rate; G = animal group (lizard vs snake); R = reproductive mode (oviparous vs viviparous); 1/kT = 1/temperature
