# Supplementary material for: Evolutionary transitions in body plan and reproductive mode alter maintenance metabolism in squamates
Source: BMC Evol Biol. 2018 Apr 3;18:45. doi: 10.1186/s12862-018-1166-5 (PMC5883405; doi:10.1186/s12862-018-1166-5)
Supplement: Supplementary file 4 — Table S4. ANOVA output of the PGLS model {ln(mSMR) ~ R × G × T} evaluating the effects of reproductive mode (R), animal clade (G) and temperature (T) on mass-corrected standard metabolic rate (mSMR). (DOC 38 kb) [file 12862_2018_1166_MOESM4_ESM.doc]

**Table S4** ANOVA output of the PGLS model {ln(mSMR) ~ R × G × T} evaluating the effects of reproductive mode (R), animal clade (G) and temperature (T) on mass-corrected standard metabolic rate (mSMR)

| Source | *df* | SQ | MSQ | *F* | *P*-value |
| --- | --- | --- | --- | --- | --- |
| Reproductive mode | 1 | 0.012 | 0.012 | 4.36 | 0.038 |
| Animal group | 1 | 0.006 | 0.006 | 3.99 | 0.047 |
| Temperature | 1 | 0.049 | 0.049 | 17.21 | < 0.001 |
| R × G | 1 | 0.005 | 0.005 | 1.87 | 0.173 |
| R × T | 1 | 0.005 | 0.005 | 1.61 | 0.206 |
| G × T | 1 | <0.001 | <0.001 | 0.02 | 0.891 |
| R × G × T | 1 | 0.008 | 0.008 | 2.74 | 0.100 |
| Residuals | 163 | 0.459 | 0.003 |  |  |
