# Supplementary material for: Evolutionary transitions in body plan and reproductive mode alter maintenance metabolism in squamates
Source: BMC Evol Biol. 2018 Apr 3;18:45. doi: 10.1186/s12862-018-1166-5 (PMC5883405; doi:10.1186/s12862-018-1166-5)
Supplement: Supplementary file 5 — Table S5. Results of the phylogenetic path analyses, ranking the candidate models according to their CICc values. The models with ΔCICc < 2 are in bold. (DOC 43 kb) [file 12862_2018_1166_MOESM5_ESM.doc]

**Table S5** Results of the phylogenetic path analyses, ranking the candidate models according to their CICc values. The models with ΔCICc < 2 are in bold

| Model | *k* | *q* | *C* | *P* | CICc | ΔCICc | *w*i |
| --- | --- | --- | --- | --- | --- | --- | --- |
| **five** | **4** | **11** | **3.980** | **0.859** | **26.340** | **0** | **0.277** |
| **four** | **5** | **10** | **5.874** | **0.826** | **27.249** | **0.909** | **0.140** |
| **eight** | **3** | **12** | **1.399** | **0.966** | **27.374** | **1.034** | **0.176** |
| **two** | **6** | **9** | **9.173** | **0.688** | **28.291** | **1.591** | **0.105** |
| three | 5 | 10 | 5.324 | 0.868 | 28.699 | 2.359 | 0.085 |
| six | 4 | 11 | 3.548 | 0.895 | 29.208 | 2.868 | 0.066 |
| one | 6 | 9 | 8.672 | 0.731 | 27.790 | 3.450 | 0.049 |
| seven | 3 | 12 | 3.343 | 0.765 | 30.317 | 3.977 | 0.038 |
| nine | 3 | 12 | 3.347 | 0.764 | 30.322 | 3.982 | 0.038 |

*k* = number of independence claims; *q* = number of parameters; *C* = Fisher’s *C* statistics; CICc = C-statistic Information Criterion; ΔCICc, difference in CICc from the best-fitting model; *w*i, CICc weights
