# Supplementary material for: Evolutionary transitions in body plan and reproductive mode alter maintenance metabolism in squamates
Source: BMC Evol Biol. 2018 Apr 3;18:45. doi: 10.1186/s12862-018-1166-5 (PMC5883405; doi:10.1186/s12862-018-1166-5)
Supplement: Supplementary file 6 — Table S6. Path statistics of the average and the best-fitting model from the phylogenetic path analyses in the squamates. For each model, the standardized regression coefficients are listed with their lower and upper 95% confidence limits. Coefficients with confidence intervals excluding 0 are in bold. (DOC 34 kb) [file 12862_2018_1166_MOESM6_ESM.doc]

**Table S6** Path statistics of the average and the best-fitting model from the phylogenetic path analyses in the squamates. For each model, the standardized regression coefficients are listed with their lower and upper 95% confidence limits. Coefficients with confidence intervals excluding 0 are highlighted in bold

| Path | Top model | |
| --- | --- | --- |
| M → SMR | **1.241** | **[0.653 / 1.829]** |
| SMR → G | **0.586** | **[0.429 / 0.743]** |
| SMR → R | **0.944** | **[0.866 / 1.022]** |
| 1/kT → SMR | **‒0.681** | **[‒0.857 / ‒0.505]** |
| 1/kT → R | ‒0135 | [‒0.331 / 0.061] |
| 1/kT → M | ‒0.196 | [‒0.412 / 0.020] |

M = body mass; SMR = standard metabolic rate; G = animal group (lizard vs snake); R = reproductive mode (oviparous vs viviparous); 1/kT = 1/temperature
